# Alpha-Lipoic Acid as an Antioxidant Strategy for Managing Neuropathic Pain

**DOI:** 10.3390/antiox11122420

**Published:** 2022-12-08

**Authors:** Max Denisson Maurício Viana, Pedro Santana Sales Lauria, Alyne Almeida de Lima, Luiza Carolina França Opretzka, Henrique Rodrigues Marcelino, Cristiane Flora Villarreal

**Affiliations:** 1 School of Pharmacy, Federal University of Bahia, Salvador 40170290, BA, Brazil; 2 Gonçalo Moniz Institute, Oswaldo Cruz Foundation, Salvador 40296710, BA, Brazil

**Keywords:** alpha-lipoic acid, antioxidant, oxidative stress, neuropathy, neuropathic pain

## Abstract

Neuropathic pain (NP) is the most prevalent and debilitating form of chronic pain, caused by injuries or diseases of the somatosensory system. Since current first-line treatments only provide poor symptomatic relief, the search for new therapeutic strategies for managing NP is an active field of investigation. Multiple mechanisms contribute to the genesis and maintenance of NP, including damage caused by oxidative stress. The naturally occurring antioxidant alpha-lipoic acid (ALA) is a promising therapeutic agent for the management of NP. Several pre-clinical in vitro and in vivo studies as well as clinical trials demonstrate the analgesic potential of ALA in the management of NP. The beneficial biological activities of ALA are reflected in the various patents for the development of ALA-based innovative products. This review demonstrates the therapeutic potential of ALA in the management of NP by discussing its analgesic effects by multiple antioxidant mechanisms as well as the use of patented ALA-based products and how technological approaches have been applied to enhance ALA’s pharmacological properties.

## 1. Introduction

Neuropathic pain (NP) is an umbrella term that defines chronic conditions marked by functional alterations of the somatosensory system due to injuries or pathological disorders. These painful conditions are heterogeneous and affect different areas of the nervous system resulting in several clinical manifestations [1]. As chronic painful conditions, including NP, have a prominent central sensitization component, they are often irresponsive to pharmacological treatments with conventional analgesic drugs since their onset [2]. Aside from the lack of effectiveness to promote satisfactory levels of analgesia, currently available drugs for treating NP are also often limited by adverse effects that some patients cannot tolerate [3]. This points out to the need for new and well-tolerated analgesic drugs that are effective in the management of NP.

Although the exact mechanisms leading to pain of neuropathic origin are still unknown, a growing body of evidence associates NP with oxidative stress [4,5]. Oxidative stress results from an imbalance between the production of free radicals and the body’s ability to eliminate those radicals by antioxidant mechanisms [6]. During NP, a failure in these mechanisms promotes a pro-oxidative microenvironment in the site of neural damage, thus compromising somatosensory signaling.

As oxidative stress plays an important role in the pathophysiology of NP, natural products and preparations rich in antioxidants have been used for treating NP with the strongest level of evidence for diabetic polyneuropathies [7]. Among these compounds, alpha-lipoic acid (ALA) is of particular interest because many studies have successfully shown its therapeutic benefits and its ability to control NP-related symptoms [7,8]. Antioxidant compounds act on different targets and pathways that ultimately contribute to improve nerve dysfunction by withstanding oxidative damage. It has been suggested that ALA has multiples antioxidant mechanisms, including metal chelating, regenerating endogenous antioxidants such as vitamins C and E, as well as modulating several signaling pathways [9].

Considering the therapeutic potential of ALA for treating NP, this review explored the antioxidant effects of ALA on pathways associated with oxidative stress in different painful conditions of neuropathic origin. This review addresses both preclinical and clinical trials, which demonstrate the analgesic potential of ALA mainly in the cases of diabetic polyneuropathies. Additionally, a patent survey was carried out to discuss the use of ALA-based products and how technological approaches have been applied to enhance ALA’s pharmacological properties.

## 2. Neuropathic Pain

NP is the most prevalent and debilitating form of chronic pain. Epidemiological studies conducted in different countries estimate that 6–10% of the general population suffer with NP [10]. A cohort survey showed that approximately 21% of patients with clinical neuropathy present symptoms of NP; this rate increases to 60% in cases of severe neuropathies [11]. Different types of NP include trigeminal neuralgia, painful polyneuropathy, chemotherapy-induced neuropathic pain, and diabetic neuropathy, all of which share common features [12]. Clinically, NP is marked by spontaneous and diffuse pain as well as by sensory-perceptual alterations. Paradoxical sensory experiences (hypersensitivity and hyposensitivity) are common in NP and other neurological disorders, making a differential diagnosis necessary. Quantitative sensory tests, questionnaires, and biopsies, among other methods, are frequently used to better characterize these symptoms and support a correct diagnosis. Different perceptual alterations are indicative of damage in distinct components of the nervous system. For example, while disturbances in the perception of vibratory and/or mechanical stimuli suggest damage in larger afferent fibers or in the dorsal column, alterations in the perception of thermal stimuli indicate injury of smaller fibers or central pain-processing pathways [12].

The management of NP is more challenging compared to other painful conditions for a number of reasons. NP is often refractory to available pharmacological treatments. First-line treatments include tricyclic antidepressants (e.g., amitriptyline), serotonin and noradrenaline reuptake inhibitors (e.g., duloxetine), and antiepileptics (e.g., pregabalin and gabapentin) [13,14]. Importantly, the available drugs for treating NP do not promote disease-modifying effects but are rather used for symptomatic treatment. Furthermore, these drugs are frequently limited due to their poor efficacy or unacceptable side effects. Along with limitations in the pharmacological sphere, NP is influenced not only by physical factors, but also by psychological and emotional aspects [15]. It compromises patients’ quality of life by affecting their productivity at work, social interactions, and family life. Moreover, NP represents a heavy financial burden on patients and health services worldwide [16,17,18].

The various and still poorly understood mechanisms of NP make it difficult to establish effective treatment protocols. As a result, many patients do not achieve adequate pain control. Studies in the field of pain have advanced significantly in the last decades, providing more robust information about NP and its pathophysiology. Recent studies have revealed a new aspect of the pathophysiology of NP that might be a keystone to guide the development of new strategies for pain management: the pivotal role of oxidative stress in NP of different origins. It has been shown that peripheral nerve damage results in significantly increased production of reactive oxygen species (ROS) and reactive nitrogen species (RNS). Therefore, nitro-oxidative stress plays an important role in neural tissue injury resulting in NP [19].

## 3. Oxidative Stress and Neuropathic Pain

Oxidative events are important for several physiological and metabolic pathways. For instance, pro-oxidative chemical entities like hydrogen peroxide and nitric oxide play a role in inflammatory signaling and vasodilation [20]. Nevertheless, maintenance of the redox homeostasis is essential in order to avoid oxidative damage. The endogenous defense system against oxidative damage includes enzymatic antioxidants such as superoxide dismutase (SOD), catalase, and glutathione peroxidase, as well as non-enzymatic small molecules like ascorbate, glutathione, flavonoids, tocopherol, carotenoid, and ubiquinol [21]. When the endogenous regulatory mechanisms are not enough to counteract oxidative events, the redox homeostasis is disrupted, and ROS build up leads to oxidative stress.

Oxidative stress is present in the pathophysiology of many neuropathic painful syndromes as a root cause, an outcome, or both. Either by initiating or maintaining the pathophysiological processes that lead to NP, the imbalance of ROS and RNS has been proposed to be central in the chain of events resulting in the neuropathic state. In fact, the nitro-oxidative stress component was associated with the development of different models of painful neuropathies, such as diabetic neuropathy [22,23], chemotherapy-induced neuropathy [24], peripheral nerve injury-induced neuropathic pain [25], and Charcot-Marie-Tooth [26]. Moreover, increased levels of oxidative stress markers were found in subacute post-stroke patients affected by NP [27]. Examples of free radicals that contribute to nitro-oxidative stress include superoxide (O_2_^•−^), hydroxyl (HO^•^), peroxyl (RO_2_^•−^), nitric oxide (NO), and nitrogen dioxide (NO_2_^•^). Nonradical species such as hydrogen peroxide (H_2_O_2_), hydrochloric acid (HOCl), nitrous oxide (HNO_2_), and peroxynitrites (R-ONOO^−^) also contribute to nitro-oxidative stress [28].

ROS modulate pain processing in different ways. They can act as second messengers to increase nociceptive transmission and/or modify the physiological environment amplifying tissue damage. Peroxynitrite, for instance, can both play a part in TRPV1 nociceptive signaling and contribute to mitochondrial toxicity in nociceptive primary afferent axons [29,30]. Additionally, evidence shows that activation of the purinoreceptor P2X7 triggers ROS production and evokes pain-like behaviors in mice [31]. A wide range of molecules, enzymes, transcription factors, and other mediators are involved in redox homeostasis. Among these components, the mitochondrial respiratory chain as well as enzymes NADPH oxidases (Nox) are important sources of ROS implicated in nociceptive sensitization/transmission during NP [20,32].

### 3.1. Mitochondrial Dysfunction and Pain Processing

Mitochondrial dysfunction is a hallmark in the pathophysiology of different types of painful neuropathy. Even under physiological conditions, mitochondria are sources of ROS within cells since electrons can escape the electron transport chain and react with oxygen to generate ROS [21]. Upon chemical aggression by chemotherapy drugs [33] or persistently elevated glucose levels [34], mitochondrial function can be affected, leading to excessive levels of ROS.

Oxaliplatin, a widely used chemotherapy agent, can induce mitochondrial atypia that is related to sensory alterations in mice [35]. Other platinum-based drugs also promote mitochondrial dysfunction, affecting cellular respiration and leading to ROS accumulation [36]. Swollen and vacuolated mitochondria were found in the nociceptive primary afferent fibers of rodents treated with oxaliplatin [35] or paclitaxel [37]. These morphologic alterations indicate a disruption in the maintenance of the proton gradient as well as impaired ATP production, characterizing a state of dysfunctional mitochondrial bioenergetics [38]. In diabetes, sustained levels of excessive glucose are associated with greater glucose metabolism. This results in increased mitochondrial membrane potential, which in turn leads to ROS overproduction and therefore the activation of pathological pathways [34].

Regardless of the cause, mitochondrial injury results in bioenergetic failure by reduced ATP production. The energy deficit affects the function of the sodium–potassium pump, leading to abnormal spontaneous firing of both Aδ and C nociceptive fibers. It also results in reduced axonal growth, ultimately leading to axon degeneration [34,39]. Another consequence of reduced ATP production via the electron transport chain is an increased dependence on glycolysis to generate energy, leading to the accumulation of lactic acid and reduced intracellular pH levels. This state also contributes to nociceptive signaling by activating receptors and channels such as TRPV1, ASIC, and P2X [40]. Finally, mitochondrial dysfunction leads to increased production of superoxide and peroxynitrite, which cause damage to proteins, lipids, and nucleic acids, hence boosting nitro-oxidative stress and contributing to neuroinflammation [22].

### 3.2. NADPH Oxidases and Pain Processing

NADPH oxidases, collectively referred to as the Nox family, are a group of transmembrane enzymes dedicated to the production of ROS. Physiologically, they can take part in post-translational processing, cellular signaling, regulation of gene expression, and cell differentiation. They also play critical roles in cardiovascular regulation and innate immunity [41]. Nevertheless, the Nox family can also be involved in pathological conditions including neurodegeneration, epilepsy [42], thrombosis [43], and chronic pain [32]. Isoforms Nox1, Nox2, and Nox4 are involved in pain processing and several studies point to their functional role on the pathophysiology of NP [32].

ROS signaling mediated by Nox1 and Nox2 contribute to the cellular and molecular events that result in mechanical allodynia and neuroinflammation in the partial sciatic nerve ligation model of NP [44]. Following nerve injury, recruited macrophages produce a Nox2-dependent oxidative burst that activates TRPA1 channels expressed on Schwann cells of the sciatic nerve. This leads to the production of ROS by Nox1, which then activates TRPA1 channels in the nociceptor to promote mechanical allodynia and a greater influx of macrophages to the injured nerve. Nox2-positive macrophages amplify oxidative stress and contribute to the upregulation of TNF-α, hence increasing the painful response [44,45]. Corroborating this mechanism, preclinical studies have shown that Nox2-deficient mice submitted to the sciatic nerve ligation model of NP develop less mechanical and thermal hypernociception and exhibit reduced production of ROS and proinflammatory cytokines as well as reduced microglia activation [32].

Nox4 has been implicated in different types of NP. This isoform is responsible for promoting TNF-α signaling [46], inducing apoptosis in dorsal root ganglia (DRG) neurons [47], and upregulating TRPA1 in the spinal cord of oxaliplatin-induced neuropathic mice [48]. Numerous studies have shown that Nox4 is involved in different mechanisms that contribute to neuropathic pain [32], but not to acute nociceptive or inflammatory pain [49]. Nox4 plays a part in structural changes and in the degradation of peripheral myelin proteins in the injured nerve [49]. Inhibition of Nox4 restores the expression of peripheral myelin proteins and ameliorates sensory alterations in neuropathic diabetic mice [50]. Pain processing in painful diabetic neuropathy models is also modulated by isoform Nox2. Upregulated levels of Nox2 have been found in both type 1 [51] and type 2 [34,52] models of painful diabetic neuropathy. Pain-related behaviors in those models were reduced upon treatments with a Nox2 inhibitor [51] and a ROS scavenger [52], respectively.

In summary, several pathways that are involved in the control of redox homeostasis are affected during NP, leading to nitro-oxidative stress. Therefore, molecules that compose these pathways are potential pharmacological targets for the treatment of painful neuropathic conditions.

## 4. Antioxidant Potential of Alpha-Lipoic Acid in Neuropathic Pain

Considering the role of oxidative stress in the pathophysiology of NP, exogenous antioxidant molecules have been assessed for therapeutic potential in animal models of painful neuropathy. The polyphenolic compound curcumin ameliorates painful diabetic neuropathy by inhibiting Nox-mediated oxidative stress in the spinal cord [51]. Lycopene has been shown to reduce pain-like behaviors of rodents in different models of NP [53,54]. Resveratrol prevents oxaliplatin-induced mechanical and thermal allodynia by both antioxidant and anti-inflammatory mechanisms [55]. Similarly, acetyl-L-carnitine, vitamin C, and ALA inhibit oxaliplatin-induced hyperalgesia [56]. Among these natural antioxidant compounds, ALA is one of the most extensively studied, standing out due to its noteworthy effects. In fact, some authors referred to ALA as “the universal antioxidant” [57]. Several factors contribute to the successful use of ALA in preclinical and clinical trials, especially for treating painful disorders [7]. The unique chemical features of ALA allow it to act by both intra- and extracellular mechanisms, unlike the majority of antioxidant compounds. Moreover, ALA effectively contraposes many pathways of oxidative stress taking place in nociceptive fibers [8,58].

### 4.1. Chemical Properties and Pharmacokinetics

**Figure 1:**
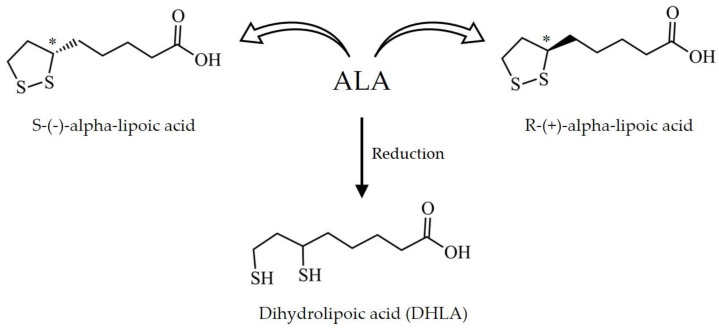
Chemical structure of alpha-lipoic acid (ALA). ALA exists as the levorotatory isomer S-(−)-ALA and the dextrorotatory isomer R-(+)-ALA. Upon reduction, ALA generates dihydrolipoic acid (DHLA). * Carbon chiral center.

As shown in Figure 1, ALA is an organosulfur medium-chain fatty acid that can be found in both oxidized (disulfide) and reduced (dithiol) forms, the latter named dihydrolipoic acid (DHLA). In its oxidized form, ALA has one carbon chiral center that results in two optical isomers: R-(+)-ALA and S-(−)-ALA. Although racemic mixtures containing equal proportions of both enantiomers are used in commercially available products based on ALA, the two isomers greatly differ in terms of their natural occurrence, biological activity, and bioavailability [59,60]. While the levorotatory form of ALA (S-(−)-ALA) is only obtained by synthetic chemistry techniques, the dextrorotatory form R-(+)-ALA occurs naturally in living organisms, including humans. This isomer is an important cofactor for diverse classes of enzymes and can be found in different tissues. It is also broadly produced by vegetable species; some of its main sources are spinach, broccoli, tomato, brussels sprouts, and rice bran [8,61,62].

The chemical characteristics of ALA strongly influence its pharmacokinetic profile. When orally administered to healthy volunteers, pure R-(+)-ALA undergoes quick and complete absorption, being the main responsible for ALA’s desirable biological effects [63]. On the other hand, the absorption of S-(−)-ALA is slower and its effects are less evident. In fact, the levorotatory form interferes with the pharmacological action of the racemic mixture by binding to non-specific targets and preventing the effects of R-(+)-ALA [63,64]. Due to its amphipathic nature, ALA can cross the blood–brain barrier. It can cross membranes through medium-chain fatty acid transporters, H^+^-linked monocarboxylate transporters, and Na^+^-dependent vitamin transport systems [65].

Despite its therapeutic potential, the limited oral absorption of ALA as a racemic mixture is a challenge to be overcome. Pharmacokinetic studies have shown that the oral bioavailability of ALA is only 30% [63,66]. As ALA competes with nutrients for sites of absorption, food ingestion is a determinant factor for ALA’s bioavailability. In fact, pharmaceutical preparations containing racemic mixtures of ALA have shown a 2-fold increase in bioavailability compared to amounts obtained through diet [8,67,68]. Some strategies have been developed to address these limitations, including innovative formulations aiming to increase ALA’s solubility and bioavailability (e.g., lecithin matrix) [66]. Other reasons as to why ALA’s oral bioavailability is low are first-pass metabolism and chemical instability in the intestinal alkaline pH [63,66].

ALA can promote biological effects in both intra- and extracellular spaces. After reaching the systemic circulation, a large amount of ALA is reduced to DHLA, which is then widely distributed to various tissues [69]. This reaction is catalyzed by enzymes that are closely related to the maintenance of redox balance, such as lipoamide dehydrogenase, thioredoxin reductase, and glutathione reductase [65]. Among other cell types, a reduction in ALA takes place in erythrocytes and involves the consumption of glucose molecules, which partially explains its effects in diabetic neuropathy [70]. Other mechanisms by which ALA counteracts NP will be discussed in the next section. Lastly, after promoting their pharmacological effects, ALA and its metabolites are mainly eliminated by renal excretion [71].

### 4.2. Pre-Clinical In Vitro and In Vivo Studies

The therapeutic use of ALA in the treatment of NP was first proposed in the end of the 1950s [72]. Since then, its antioxidant properties have been extensively studied both in vitro and in vivo. These studies have contributed to our knowledge on ALA and its analgesic effects by multiple antioxidant mechanisms. ALA modulates oxidative stress by acting as a free radical scavenger, metal chelator, and detoxifying agent. It regenerates endogenous antioxidants, diminishes lipid peroxidation, and modulates various signaling pathways, including the insulin and the NF-κB pathways [73,74,75]. ALA is also a cofactor for enzymatic complexes involved in energy generation for the cell, whose failure is observed in some types of NP [8,34,39]. Moreover, ALA improves nerve blood flow and nerve conduction of both sensory and motor signals [76,77].

Painful diabetic neuropathy is the most common type of NP. High glycemic levels and oxidative stress are key features in the pathophysiology of diabetic neuropathy [78]. The murine model of streptozotocin (STZ)-induced diabetic neuropathy is marked by oxidative stress, which is characterized by hyperglycemia followed by formation of advanced glycation end products (AGES), diminished antioxidant capacity, low glutathione levels, and mitochondrial dysfunction [79]. Oxidative stress driven by sustained hyperglycemia ultimately leads to oxidative damage and apoptosis of neuronal cells [80]. Given its good representativity of the human disease, many authors have used this model to assess the effects of ALA in diabetic neuropathy.

STZ-induced diabetic rats intraperitoneally treated with ALA (30, 60, and 120 mg/kg) have shown attenuated thermal and mechanical hyperalgesia [81]. Another set of experiments has shown that oral treatment with ALA (100 mg/kg) reduces the generation of oxidative stress biomarkers, increases the levels of glutathione, and ameliorates mitochondrial dysfunction in DRG neurons by attenuating morphological alterations and increasing ATP production [82]. Likewise, ALA (100 μM) increases the ATP/ADP ratio as well as the levels of the antioxidant enzymes SOD and catalase, which results in decreased ROS production in vitro [83].

Oral treatments with ALA (100 mg/kg) also reduce apoptosis of DRG neurons; this effect is accompanied by decreased levels of caspase 3, an important protein in apoptosis signaling [82]. Similarly, Chen and Li [80] have demonstrated that STZ-induced diabetic rats intraperitoneally treated with ALA (100 mg/kg) show a reduced sciatic nerve cell apoptosis index when compared to non-diabetic control rats. This protective effect is possibly due to the inhibition of the enzyme poly (ADP-ribose) polymerase (PPAR), another apoptosis mediator. Corroborating these results, in vitro assays performed on neuroendocrine cells exposed to high glucose levels have shown that ALA (100 μM) inhibits the expression of the apoptosis markers Bax, Bcl-2, and caspase 3 [83]. The relationship between oxidative stress and apoptosis during NP had been previously demonstrated by Siniscalco et al. [84], who showed that ROS regulates the expression of apoptotic genes in the chronic constriction injury model of NP in mice.

In vitro experiments performed by Jain and Lim [85] have shed light on a potential mechanism by which ALA can delay or inhibit the development of diabetic neuropathy. The authors have shown that human red blood cells exposed to high concentrations of glucose and treated with ALA (100 and 200 μM) have a significant increase in glucose metabolism as well as reduced levels of glycated hemoglobin. Additionally, ALA reduces the levels of lipid peroxidation markers and increases (Na^+^+K^+^)- and Ca^2+^-ATPase activities. Both glycated proteins and lipid peroxidation cause structural and functional changes on membranes. This results in inhibition of ATPase activity, disrupting the balance of electrochemical gradients and, thus, signal transduction and membrane excitability. By inhibiting these changes, ALA prevents the sensory alterations that are common in painful diabetic neuropathy.

The analgesic effects of ALA in diabetic neuropathy are not limited by its antioxidant mechanisms. ALA has also been shown to downregulate the expression of TRPV1 channels and P2X3 purinoceptors in the DRG of diabetic rats [81,86]. These receptors are responsible for the transduction of nociceptive stimuli and are involved in mechanisms of painful sensitization in the DRG [81]. In fact, inhibition of both P2X3 and TRPV1 in the DRG is associated with attenuated hyperalgesia in the rat [87,88]. ALA downregulates the expression of these receptors by blocking the NF-κB signaling pathway. This effect can either be direct or a consequence of the regeneration of vitamin E by ALA. Both ALA and vitamin E modulate upstream enzymes responsible for the disinhibition of NF-κB, thereby preventing its activation [89]. Avoiding NF-kB activation is possibly the mechanism by which ALA has been shown to reduce neuroinflammation markers in vitro [90], considering that NF-kB is an important transcription factor associated with glial activation and transcription of pro-inflammatory cytokines such as TNF-α and IL-1β, which lead to spinal neuroinflammation and increased ROS production [91].

Several studies have shown the consistent antinociceptive effects of ALA in murine models of diabetic neuropathy. Nevertheless, the therapeutic properties of ALA have also been characterized in other experimental models of NP. Wang et al. [92] evaluated the effects of ALA (50 mg/kg) on the peripheral nerve constriction model of NP. Treatments prevented morphological changes and apoptosis of DRG neurons. The proposed mechanism was the reduction in satellite glial cells and P53+ cells, involved in the initiation of neuropathic pain and apoptosis, respectively. Khan et al. [93] studied the effects of subcutaneous ALA (10 mg/kg) on a mouse model of autoimmune encephalomyelitis that mimics multiple sclerosis-associated NP. Daily treatments with ALA increased mechanical nociceptive thresholds after three weeks. Additionally, ALA treatments reduced CD3+ T-cell infiltration, microglia activation, and BDNF-TrkB signaling in the dorsal horn of the spinal cord.

Melli et al. [94] tested the effects of ALA in an in vitro model of chemotherapy-induced peripheral neuropathy. This model consists in exposing primary cultures of sensory neurons of DRG of rats to chemotherapy agents (paclitaxel and cisplatin), causing morphological and functional alterations characteristics of neuropathy. The authors have shown that pre-incubation with ALA (50 mg/mL) prevents apoptosis, axonal degeneration, and mitochondrial dysfunction indicated by the presence of vacuoles. The effects of ALA in this model are due to its antioxidant properties, especially due to the induction of frataxin expression, an essential protein for mitochondrial function.

**Figure 2:**
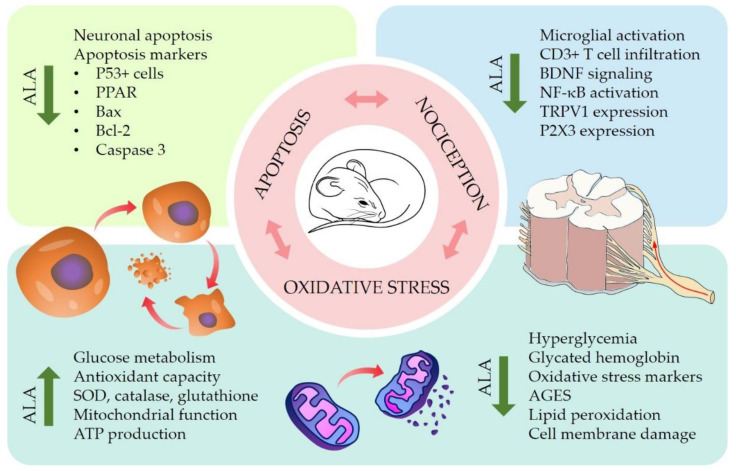
Effects of alpha-lipoic acid (ALA) on preclinical models of neuropathic pain (NP). In vitro and in vivo studies have provided insights in the mechanisms by which ALA reduces neuronal apoptosis, oxidative stress, and nociceptive signaling in several models of NP. Graphical representations of mouse and spinal cord are adapted with permission from SciDraw.io [95,96].

Preclinical studies have revealed the therapeutic potential and possible mechanisms of ALA in the treatment of different types of NP. ALA promotes protective effects against the oxidative stress, pathological alterations, and apoptosis triggered by hyperglycemia. It also increases the levels of endogenous antioxidants such as glutathione and SOD, thereby restoring redox homeostasis and maintaining the integrity of the mitochondrial membrane. In vivo experiments have shown that treatments with ALA reduce pain-like behaviors in different murine models of NP by modulating transcriptional pathways involved in the sensitization of nociceptive neurons. The results discussed in this review support the promising use of ALA as a therapeutic agent for the management of NP. The antioxidant mechanisms of ALA contributing to its antinociceptive effects on NP are summarized in Figure 2.

### 4.3. Clinical Trials

The promising results of preclinical investigations have been confirmed by successful clinical trials conducted since the 1990s. These studies have shown that ALA promotes significant improvement in the clinical condition of patients with NP. A systematic review with quantitative meta-analysis performed by Mijnhout et al. [97] concluded that intravenous treatment with ALA (600 mg/day) for three weeks significantly reduced the symptoms of peripheral diabetic neuropathy in patients that were irresponsive to other therapies, although photosensitivity is a limiting factor for the use of intravenous ALA. The effect of ALA was considered clinically relevant and surprisingly fast when compared to other antioxidant supplements. Although oral treatments with ALA at doses ≥ 600 mg/day have also significantly reduced the symptoms of painful diabetic neuropathy, it is not clear whether this improvement can be considered clinically relevant [97]. The limitations of oral treatments can be associated with the small oral bioavailability of ALA, as discussed in Section 4.1.

Despite the drawbacks of the oral route, there are studies in which oral treatments with ALA promote effective and clinically relevant analgesia. Kulakli et al. [98] report the case of a woman that suffered from multiple sclerosis-associated NP refractory to first-line drugs (amitriptyline, pregabalin, and gabapentin). The patient was given daily oral treatment with ALA (600 mg/day) and showed significant improvements after three weeks. She reported lower pain scores and a general improvement in her quality of life, as assessed by the LANSS Pain Scale and the Short Form Health Survey, respectively. After two months of treatment, the patient reported that she could wear socks again for the first time in three years, for she no longer felt the burning pain caused by the touch of the fabric on her feet.

A recent review conducted by Ziegler et al. [99] corroborates the use of intravenous ALA (600 mg/day) to improve the symptoms of painful neuropathy. The authors also recognize the potential of ALA in the treatment of diabetic sensorimotor polyneuropathy (DSPN). This is according to a clinical trial involving 460 diabetic patients with mild-to-moderate DSPN. ALA improved the natural history of DSPN and was well-tolerated by patients during the four-year period of the study. Thus, ALA was considered effective and safe in the management of DSPN and is currently one of the few therapeutic agents approved for the treatment of this condition in humans in several countries. Finally, Ziegler et al. [99] also reported improvements in biochemical markers of microvascular damage in patients with type 1 diabetes after four weeks of combined treatment with benfotiamine (300 mg) and ALA (600 mg) twice daily.

Only one clinical study was found on the clinicaltrials.gov database using the keywords “alpha-lipoic acid” and “neuropathic pain”. This study (identifier NCT03428139) evaluates the use of ALA as an adjuvant therapy combined with pulsed radiofrequency for the treatment of chronic lumbosacral radicular pain. The results of this research have been recently published [100]. Patients with lumbo-sacral radicular pain were treated either with pulsed radiofrequency alone or with pulsed radiofrequency combined with oral administration of ALA (600 mg in variable dosing intervals). The study demonstrated superior results in the group that received ALA as adjuvant therapy. Different methods were used to score pain levels of patients; all of them showed a significant decrease in pain when assessed three and six months after the beginning of the study. These results were associated with ALA’s ability to improve nerve function and inflammation.

Other clinical studies involving the therapeutic use of ALA can be found on the clinicaltrials.gov database. Most of these works are phase 1 and phase 2 trials conducted in the United States and Egypt. In these trials, ALA is rarely used alone, but rather combined with other drugs and dietary supplements. These studies do not address the use of ALA for managing NP and were not included in this review.

## 5. Innovation and Alpha-Lipoic Acid-Based Products

To assess the extent of ALA research for therapeutic purposes, various sources of information, patents, and original research articles were examined. There was a trend towards an increase in the number of patents filed and granted that focused on ALA formulations for various therapeutic purposes. New therapeutic strategies were also examined in original research articles that describe several approaches for developing ALA formulations, including metal-based, polymer-based, and lipid-based nanoparticles. These new strategies aimed to enhance the therapeutic properties of ALA by improving its biopharmaceutical and pharmacokinetic profile.

### 5.1. Patents

**Figure 3:**
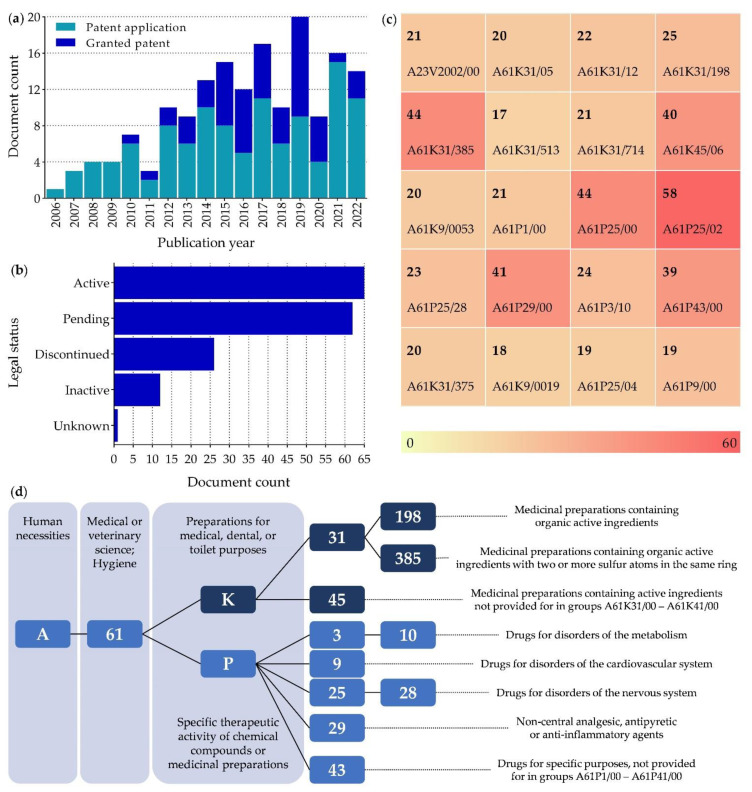
Summary of patent data on “alpha lipoic acid”, “neuropathic pain”, and “oxidative stress”. (**a**) Granted patents and patent applications over the years; (**b**) Legal status of the patents in July, 2022; (**c**) Most commonly used Cooperative Patent Classification (CPC) for patents in alphanumeric order. Heat map shows the frequency of their appearance on the search performed; (**d**) Description of the 10 most frequently used CPC.

To evaluate the innovation and commercial interest in ALA, a search was performed in the lens.org database in July 2022 using the keywords “alpha lipoic acid” AND “neuropathic pain” AND “oxidative stress”. The search performed limited the number of formulations/compositions for which patents were filed after excluding studies on the use of ALA in other diseases. Nevertheless, this search yielded a total of 167 patents belonging to 78 simple families. Most of these patents were filed and issued in the decade between 2011 and 2020 and are still active. However, the number of pending patents is almost as high as the number of granted patents (Figure 3). The United States is the country with the most pending patents (110 United States, 45 World Intellectual Property Organization, and 12 European Patent Office), which agrees with the fact that the United States is one of the few countries where ALA-based products are available on the market, as discussed below in Section 5.2.

The most used Cooperative Patent Classification (CPC) codes (Figure 3) are for the treatment of diseases affecting the nervous system, as expected from the used keywords, but also cardiovascular diseases, which can be due to the association between cardiovascular diseases and the inflammatory response [101]. Most patents focus on combining ALA with other drugs in pharmaceutical preparations. However, there are only a few that focus on the use of cyclodextrins and liposome-loaded nanogels to improve ALA pharmacological properties.

### 5.2. Market

In the rational design of this work, the first step was searching for clinical trials, then for patents, and finally for products already on the market. For this last step, the go.drugbank.com database was used. However, since ALA is not a drug, only formulations with ALA in combination with drugs were found. To our knowledge, there is no database for approved dietary supplements, where most ALA products should be found.

Only the United States and Turkey have products containing ALA on the market. Interestingly, the United States has the majority of patents for preparations/formulations containing ALA, while Turkey is not mentioned on the lens.org database. The products are only available as oral dosage forms (coated tablets: enteric-coated and modified-release). The three topical products previously available were withdrawn from the market in both countries. Additionally, associations of ALA with other molecules in the same dosage form were found to be consistent with the profile observed in the clinical studies (Section 4.3). The most frequent associations of ALA were with metformin, pioglitazone, and cyanocobalamin. It is also noteworthy that ALA is available in two dosages, 300 mg and 600 mg, indicating a wide therapeutic window for its use. Finally, most releases occurred in 2020, likely due to an anti-inflammatory effect in the treatment of inflammation caused by the SARS-CoV-2 virus, as suggested in patent deposit US 202117366007 A.

## 6. Novel Therapeutic Strategies

**Figure 4:**
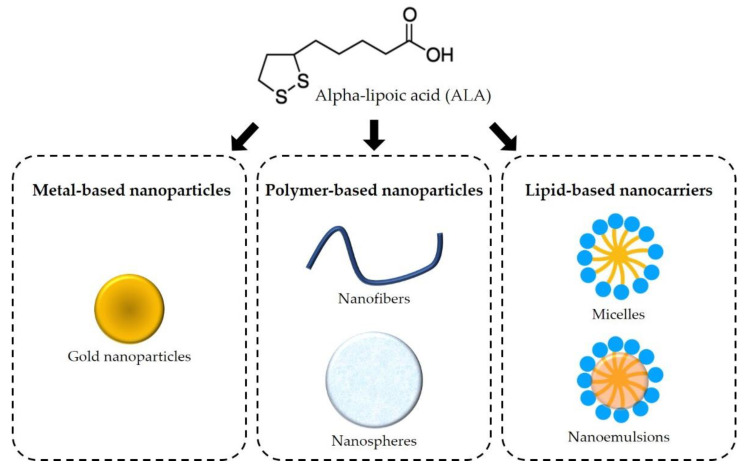
Schematic of the main nanotechnological approaches using alpha-lipoic acid.

In addition to the regular dosage forms already on the market, the use of nanocarriers has been investigated to enhance the biological effects of ALA, which are greatly influenced by ALA’s low solubility in the gastrointestinal tract and extensive hepatic degradation during first-pass metabolism [8]. In the last five years, several approaches have been investigated, including metal-based, polymer-based, and lipid-based nanoparticles (Figure 4). These novel pharmaceutical products will be discussed in this section.

### 6.1. Metal-Based Nanoparticles

The most common metal-based nanotechnology approach to improve ALA delivery is conjugation with gold nanoparticles (GNP). However, in the publications that investigated this technology, the nanocarriers were not evaluated in vivo. Crescenzo et al. [102] studied the synthesis of GNP with sodium borohydride (NaBH_4_). Later, they capped the nanoparticles with L-dopa-ALA or dopamine-ALA prodrugs. GNP synthesis was optimized, and the L-dopa-ALA formulation was evaluated for drug release and toxicity using the 3-(4,5-dimethylthiazol-2-yl)-2,5-diphenyltetrazolium bromide (MTT) assay for cell viability. The optimized formulation released the entire drug content within 1 h and showed good biocompatibility with the SH-SY5Y cell line, a human neuronal cell model for Parkinson’s disease research. These data suggest that metal-based nanoparticles are a promising technology that needs further investigation.

Additionally, Piersimoni et al. [103] synthesized GNP capped with ALA and studied their antioxidant activity in an in vitro model of Parkinson’s disease using SH-SY5Y cells. First, they examined the GNP morphologically to verify the presence of ALA and alpha-succinate, a model molecule that causes oxidative stress. Changes were observed in average particle size (18 to 23 nm), polydispersity index (0.2 to 0.3), zeta potential (−13.6 to −53.6 mV), and atomic force microscope response, indicating successful binding. Later, cytotoxicity was assessed with the MTT assay to determine the concentrations required to achieve no cytotoxic effect of GNP and GNP-ALA. GNP-alpha-synuclein were also evaluated for their ability to induce oxidative stress. They successfully induced alpha-synuclein aggregation and consequently the appearance of the toxic effects on SH-SY5Y cells. Subsequently, the cells exposed primarily to GNP-alpha-synuclein were exposed to GNP-ALA. The concentrations found to be biocompatible for GNP-ALA were used to assess mitochondrial activity, measure lipid peroxidation, and perform structural analysis. In conclusion, GNP-ALA protect living cells’ SH-SY5Y by antioxidant mechanisms against ROS damage and protect the microtubular structure of the cell, maintaining the biophysics of the cell membrane. Despite the successful results, in vivo studies must be conducted with caution since the continuous administration of GNP, especially at high doses, can lead to residual accumulation and toxicity.

### 6.2. Polymer-Based Nanoparticles

Approaches using polymeric matrices have also been evaluated. Some examples are nanospheres and nanofibers prepared with sustained-release polymers, such as polycaprolactone and poly(lactic-co-glycolic acid). Aljaeid and El-Moselhy [104] used ALA as a biologically active molecule to reduce the nephrotoxicity of gentamicin. To achieve this goal, these two molecules were encapsulated into the nanoparticles using a nanoprecipitation technique, morphologically characterized, and then tested in vivo in rabbits. The prepared loaded nanoparticles were spherical and had a diameter of about 540 nm. Gentamicin-treated rabbits showed increased levels of biochemical markers of kidney damage compared to vehicle-treated control rabbits. On the other hand, biochemical markers of rabbits that received nanoparticles loaded with both gentamicin and ALA were not significantly different from those of the control group, indicating that ALA had a protective effect against gentamicin-induced nephrotoxicity.

Haidar et al. [105] investigated the use of poly(lactic-co-glycolic acid) nanofibers to deliver ALA and atorvastatin for treating peripheral nerve injury. Atorvastatin was preloaded in chitosan nanoparticles. These nanoparticles were loaded with ALA into the poly(lactic-co-glycolic acid) solution, which was then electrospun. The nanofibers were successfully prepared and exhibited a multi-step release profile: a one-step release profile for ALA and a two-step release profile for atorvastatin. About 83% of ALA was released within the first hour. The nanofibers were biocompatible with two cell lines (L-929 and B-35). Subsequent in vivo assays performed on male Sprague-Dawley rats (250–300 g) showed that the formulation containing ALA and atorvastatin was superior in restoring motor function compared to formulations containing only one of the active molecules, indicating a synergic effect when ALA and atorvastatin are combined.

### 6.3. Lipid-Based Nanocarriers

The lipid-based approaches applied to ALA include the generation of micelles and nanoemulsions. Kulikova et al. [106] developed a patent-protected micellar system by complexing ALA and carnosine. The neuroprotective effect of this formulation was tested in a rat model of neurotoxicity induced by 1-methyl-4-phenyl-1,2,3,6-tetrahydropyridine (MPTP). The formulation containing ALA and carnosine promoted a superior neuroprotective effect when compared to carnosine alone, as assessed by the levels of lipid peroxidation as well as catecholamines and their metabolites. The micellar system reestablished the antioxidant activity in the brain tissue and the metabolism of dopamine and 5-hydroxytryptamine, suggesting a potential use in the treatment of Parkinson’s disease.

Another micellar system was developed by Kobuta et al. [107] by mixing ALA and stearyl poly(20)oxyethylene ether and then coating the micelles with inorganic metal salts (magnesium and carbonate). The resulting nanostructures were dispersible in water and had a diameter of 8–15 nm. In vivo assays performed on hairless mice and guinea pigs aiming to evaluate the anti-aging effects of the formulation showed that the micellar system had good skin permeability and dose-dependently stimulated the proliferation and differentiation of keratinocyte in the skin, caused thickening of the epidermis, and improved ultraviolet light-induced pigmentation.

Lastly, Çoban et al. [108] developed and evaluated a few nanoemulsion formulations containing ALA. These nanoemulsions were prepared with castor oil or sunflower oil by different stirring methods (magnetic stirring or Ultra-Turrax) and contained ALA and cyanocobalamin combined. The effects of the multiple combinations on the physicochemical properties and release profile of ALA were studied. The physical and chemical stability of the formulations at different temperatures (30 °C, 50 °C, and 70 °C) and pH levels (1.2, 4.5, 6.8, and 7.4) were evaluated and ALA was quantified by HPLC. The analyses revealed that the castor oil-based formulation prepared by the solvent displacement method under magnetic stirring was the most stable formulation under all conditions studied. The ALA and cyanocobalamin content of the formulation was almost completely released after five hours; cyanocobalamin showed a slightly slower release. No pharmacological tests were performed with the nanoemulsions developed.

## 7. Conclusions

ALA is a bioactive molecule that promotes relevant therapeutic effects. The antioxidant properties of ALA endorse its potential for the treatment of NP. Several preclinical and clinical studies support the use of ALA in the management of neuropathic painful conditions. Preclinical in vitro and in vivo assays demonstrate that ALA modulates different pathways of oxidative stress and attenuates nociception in NP models of diabetic neuropathy, peripheral nerve constriction injury, and autoimmune encephalomyelitis mimicking multiple sclerosis-associated pain. Clinical studies corroborate the analgesic effects of ALA by antioxidant mechanisms in patients with painful diabetic neuropathy, neuropathic pain associated with multiple sclerosis, diabetic sensorimotor polyneuropathy, and chronic lumbosacral radicular pain.

Despite its therapeutic benefits, some barriers must be overcome before the approval of clinical use of ALA in the management of NP. Clinical trials lack well-standardized treatment protocols including doses and dosing intervals. Moreover, many commercially available products associate ALA with other compounds, especially antidiabetic drugs, making it difficult to assess the effect of ALA as a monotherapy. Lastly, the limited pharmacokinetic profile of ALA is a major limitation of its use. Although novel pharmaceutical technologies have allowed the development of nanocarrier-based formulations aiming to enhance ALA’s pharmacological properties, the effect of these new products on NP is unclear.

Based on successful preclinical and clinical trials, it is possible to conclude that ALA has the potential to be a complementary therapy for NP, mainly for diabetic polyneuropathies. The need for pharmacokinetic optimization of new ALA-based products has encouraged new research and investment, reflected in the growing number of patents registered in recent years. However, studies aiming the better characterization of the therapeutic use and clinical feasibility of ALA are needed.

## Data Availability

Not applicable.

## Abbreviations

ADP	Adenosine diphosphate
AGES	Advanced glycation end products
ALA	Alpha-lipoic acid
ASIC	Acid-sensing ion channels
ATP	Adenosine triphosphate
BDNF	Brain-derived neurotrophic factor
CPC	Cooperative patent classification
DHLA	Dihydrolipoic acid
DRG	Dorsal root ganglia
DSPN	Diabetic sensorimotor polyneuropathy
GNP	Gold nanoparticles
IL-1β	Interleukin 1 beta
IPC	International patent classification
MPTP	1-methyl-4-phenyl-1,2,3,6-tetrahydropyridine
MTT	3-(4,5-dimethylthiazol-2-yl)-2,5-diphenyltetrazolium bromide
NADPH	Nicotinamide adenine dinucleotide phosphate
NF-κB	Nuclear factor kappa-light-chain-enhancer of activated B cells
Nox	Nicotinamide adenine dinucleotide phosphate oxidases
NP	Neuropathic pain
PPAR	Poly (ADP-ribose) polymerase
RNS	Reactive nitrogen species
ROS	Reactive oxygen species
SOD	Superoxide dismutase
STZ	Streptozotocin
TNF-α	Tumor necrosis factor alpha
TrkB	Tropomyosin receptor kinase B
TRPA1	Transient receptor potential ankyrin subtype 1
TRPV1	Transient receptor potential vanilloid subtype 1
